# Celebrating Ochsner's 80th Anniversary Year

**DOI:** 10.31486/toj.22.5025

**Published:** 2022

**Authors:** Ronald G. Amedee

**Affiliations:** Director of Clinical School and Professor, The University of Queensland Medical School, Ochsner Clinical School, New Orleans, LA; Editor-in-Chief, *Ochsner Journal*

*Spring makes its own statement, so loud and clear that the gardener seems to be only one of the instruments, not the composer.*

*–Geoffrey B. Charlesworth*

Six original research articles, one contemporary update paper, two quarterly columns, and seven case reports and clinical observations serve as the principal elements for the Spring 2022 issue of the *Ochsner Journal*, our first issue in Ochsner's 80th anniversary year. This year, as in years past, the *Ochsner Journal* will continue its meaningful contributions to the realization of Ochsner's mission to “serve, heal, lead, educate, and innovate.”

“Opioid Use After Elective Otolaryngologic Surgery at a Teaching Institution” adds important data and observations to the critical conversation regarding the prescription of opioids and the subsequent dependency in some patients, a topic that continues to dominate the headlines. A fact sheet released by the White House on March 1, 2022, points out that drug overdose deaths have reached an historic high in this country, with more than 104,000 US citizens dying from overdoses in the 12-month period ending September 2021. Tailoring prescriptions to individual patients instead of relying on practice patterns is one approach to reducing the number of excess pills that are potentially available for diversion.

“Cutaneous Manifestations in COVID-19–Positive African American Patients” by Bryan, Samant, Asarkar, et al and “Cross-Sectional Survey of Smoking Patterns During the COVID-19 Pandemic in a Tobacco Cessation and Lung Cancer Screening Program” by Mejia, Zoorob, Levine, and others are original research manuscripts that continue to explore findings from the COVID-19 pandemic. One of the coauthors of the Mejia et al paper is Professor Charles Hennekens, a 2014 winner of the Alton Ochsner Award Relating Smoking and Disease.

“Transparency in Admissions and Personalized Learning Through Resident Patient Selection” authored by Archibald, Zimmerman, Seay, et al and “Pilot Study for Assessing Nontechnical Skills in Emergency Medicine Residents: Why We Should C.A.R.E.” by Caffery, D’Antonio, Pogue, and Musso highlight important work being done in graduate medical education at their respective institutions.

Our colleagues at LSU have compiled “A Framework for the Virtual Medical Interview Process: Considerations for the Applicant and the Interviewer,” a paper that's chock-full of tips for acing the Zoom interviews that have become common in these pandemic times.

This issue's quarterly Sports Medicine and Clinical Images columns are “Sacroiliac Joint Pain in the Athlete” from Pfeiffer, Kobayashi, and Gottschalk, and “Spinal Dural Arteriovenous Fistula: The Missing-Piece Sign” from Kelley, April, Bagert, Milburn, and Steven.

Several case reports have an orthopedic theme, and most of the coauthors are Ochsner folk—medical students, residents, and attendings: “Indoor Skydiving: An Emerging Cause of Anterior Shoulder Dislocations,” “Patellar Dislocation and Fracture After Medial Patellofemoral Ligament Reconstruction in a Patient With Osteogenesis Imperfecta,” and “Surgical Resection of Bertolotti Syndrome.” The cover art for this issue of the *Ochsner Journal* is by a 2021 graduate of Ochsner Clinical School, Daniel M. Zumsteg, who created an original illustration for the case report about an indoor skydiving injury. We are delighted to feature his work on the cover.

As a wonderful complement to Ochsner's anniversary, we received a letter from Dr Mark Hoffer, a distinguished orthopedic surgeon who did his residency at Ochsner in the 1960s and worked with founder Dr Guy Caldwell. Dr Hoffer writes on the theme of mentorship and its importance. And Dr Marianne Maumus, a gifted faculty leader of the Character in Medicine course that is taught to third-year Ochsner Clinical School students, has contributed an important editorial on the benefits of gratitude.

Finally, abstracts from the 13th Annual Evidence-Based Practice/Research Conference with the theme of “Nurses Shaping Health Care: Retaining the Nursing Workforce” are included as online-only content for this edition. Access the abstracts at the *Ochsner Journal* website: www.ochsnerjournal.org/.

I wish each of you a warm and splendid springtime which has already afforded New Orleanians and many visitors to our area a spectacular return of Mardi Gras following the 1-year hiatus caused by the pandemic, and the New Orleans Jazz & Heritage Festival is also about to make a return. Statewide, COVID numbers are trending downward, while average daily temperatures are steadily rising. Spring is almost formally here, along with daylight saving time. Please get out and enjoy this wonderful time of year while staying safe.

